# Outcomes of Postoperative Overnight High-Acuity Care in Medium-Risk Patients Undergoing Elective and Unplanned Noncardiac Surgery

**DOI:** 10.1001/jamasurg.2023.1035

**Published:** 2023-05-03

**Authors:** Guy Ludbrook, Michael P. W. Grocott, Kathy Heyman, Sandy Clarke-Errey, Colin Royse, Jamie Sleigh, L. Bogdan Solomon

**Affiliations:** 1 Central Adelaide Local Health Network, Adelaide, Australia; 2 The University of Adelaide, Adelaide, Australia; 3 Perioperative and Critical Care Research Theme, Southampton NIHR Biomedical Research Centre, University Hospital Southampton, University of Southampton, Southampton, United Kingdom; 4 Statistical Consulting Centre, The University of Melbourne, Parkville, Australia; 5 Department of Surgery, The University of Melbourne, Royal Melbourne Hospital, Parkville, Australia; 6 Outcomes Research Consortium, Cleveland Clinic, Cleveland, Ohio; 7 The University of Auckland, Peter Rothwell Academic Centre, Waikato Hospital, Hamilton West, Hamilton, New Zealand

## Abstract

**Importance:**

Postoperative complications are increasing, risking patients’ health and health care sustainability. High-acuity postoperative units may benefit outcomes, but existing data are very limited.

**Objective:**

To evaluate whether a new high-acuity postoperative unit, advanced recovery room care (ARRC), reduces complications and health care utilization compared with usual ward care (UC).

**Design, Setting, and Participants:**

In this observational cohort study, adults who were undergoing noncardiac surgery at a single-center tertiary adult hospital, anticipated to stay in hospital for 2 or more nights, were scheduled for postoperative ward care, and at medium risk (defined as predicted 30-day mortality of 0.7% to 5% by the National Safety Quality Improvement Program risk calculator) were included. Allocation to ARRC was based on bed availability. From 2405 patients assessed for eligibility with National Safety Quality Improvement Program risk scoring, 452 went to ARRC and 419 to UC, with 8 lost to 30-day follow-up. Propensity scoring identified 696 patients with matched pairs. Patients were treated between March and November 2021, and data were analyzed from January to September 2022.

**Interventions:**

ARRC is an extended postanesthesia care unit (PACU), staffed by anesthesiologists and nurses (1 nurse to 2 patients) collaboratively with surgeons, with capacity for invasive monitoring and vasoactive infusions. ARRC patients were treated until the morning after surgery, then transferred to surgical wards. UC patients were transferred to surgical wards after usual PACU care.

**Main Outcome and Measures:**

The primary end point was days at home at 30 days. Secondary end points were health facility utilization, medical emergency response (MER)–level complications, and mortality. Analyses compared groups before and after propensity scoring matching.

**Results:**

Of 854 included patients, 457 (53.5%) were male, and the mean (SD) age was 70.0 (14.4) years. Days at home at 30 days was greater with ARRC compared with UC (mean [SD] time, 17 [11] vs 15 [11] days; *P* = .04). During the first 24 hours, more patients were identified with MER-level complications in ARRC (43 [12.4%] vs 13 [3.7%]; *P* < .001), but after return to the ward, these were less frequent from days 2 to 9 (9 [2.6%] vs 22 [6.3%]; *P* = .03). Length of hospital stay, hospital readmissions, emergency department visits, and mortality were similar.

**Conclusions and Relevance:**

For medium-risk patients, brief high-acuity care with ARRC allowed enhanced detection and management of early MER-level complications, which was followed by a decreased incidence of subsequent MER-level complications after discharge to the ward and by increased days at home at 30 days.

## Key Points

**Question:**

What are outcomes of high-acuity overnight postoperative care using a highly structured advanced recovery room care (ARRC) model in medium-risk surgical patients?

**Findings:**

In this cohort study of 854 adult noncardiac surgical patients, compared with usual ward care, ARRC resulted in a statistically significant 50% decrease in in-hospital medical emergency response–level complications and 2 more postoperative days at home.

**Meaning:**

In this study, ARRC was associated with reduced major postoperative complications and increased days at home after surgery.

## Teaser

This cohort study evaluates whether a new high-acuity postoperative unit, advanced recovery room care, reduces complications and health care utilization compared with usual ward care.

## Introduction

Surgery is required for a high proportion of health care needs. However, sustainable access to surgery is challenged by the impact of populations’ increasing age and prevalence of comorbidities, translating into frequent, debilitating, and costly early postoperative complications.^1,2^ These have adverse effects on patients’ recovery^3^ and hospitals’ capacity.^4^

While the burden of complications in high-risk patients is well described,^5^ patients at medium risk are often overlooked. Medium risk usually refers to a predicted 30-day mortality of around 1% to 5%^6^ and is a rapidly growing proportion of surgical patients. Medium-risk patients are usually managed postoperatively in standard surgical wards, but it has become apparent this often results in missed early medical emergencies, such as hypotension,^7,8^ with increased risk of harm.^9^

High-acuity and enhanced postoperative facilities have been suggested to address this.^10,11^ However, such facilities are often difficult to obtain in cost-constrained health care systems when traditional high-acuity units (intensive care units [ICUs], high-dependency units, and surgical special care units) can be 5-fold the cost of a ward. Further, there is little strong scientific evidence of clinical benefit or cost-effectiveness of enhanced postoperative facilities,^12,13,14^ with high reliance on observational data. A prospective clinical trial of high-acuity care to address efficacy and cost is considered desirable,^13^ but such prospective health system trials are challenging to conduct,^12^ mainly because of concerns about clinician equipoise and patient willingness to be randomized.

A recent feasibility trial examined high-acuity postoperative care of medium-risk patients through a highly structured model, termed *advanced recovery room care* (ARRC), which extends traditional recovery room (or postanesthesia care unit [PACU]) care up to 24 hours.^8^ ARRC I data showed improved early detection of medical emergencies and possible improved clinical outcomes cost-effectively.^15,16^ To build on this, we designed an adequately powered prospective single-center cohort study of ARRC. This addressed the hypothesis that patients receiving ARRC would have a higher incidence of early medical emergency complications detected and managed early after surgery, resulting in fewer subsequent inpatient ward emergency events, and producing improved clinical outcomes and hospital utilization within 30 days. Whether this would produce net cost savings will be examined and reported separately using cost-effectiveness modeling.^16^

## Methods

### Study Design

This was an observational single-center open-label cohort study of the outcomes of early high-acuity care with ARRC until the morning after surgery. Ethical approval was received from the Central Adelaide Local Health Network Human Research Ethics Committee (approval number 14076). The requirement for written informed consent was waived. The study was conducted at the Royal Adelaide Hospital, a large adult tertiary teaching hospital, between March 1, 2021, and March 23, 2022.

### Participants

Patients studied were those undergoing elective or unplanned noncardiac surgery and scheduled for postoperative care in a standard surgical ward for at least 2 postoperative nights. Patients were identified from operating schedules, then screened prior to the start of operating lists to identify those considered medium risk, defined as predicted 30-day mortality of 0.7% to 5% by the American College of Surgeons National Safety and Quality Improvement Program (NSQIP) risk calculator.^17^

### Group Allocation

Patients from the pool of those eligible were allocated to ARRC in order of commencement of surgery until beds were full, with subsequent eligible patients receiving usual care (UC). Approximately 3 to 4 ARRC beds were usually available daily.

### Intervention

The ARRC model has been described previously, although for this study, the inclusion criteria were broadened slightly, and in-house physicians were present continuously.^8^ It uses the capacity usually available in a PACU and extends care to the morning after surgery and is described in detail in eAppendix 1 in Supplement 1. In brief, it has a nursing ratio of 1 nurse to 2 patients, on-site anesthesia-experienced physicians, continuous monitoring (including invasive monitoring), and capacity for a broad range of intravenous fluids and medicines, including low-dose vasopressors. No positive pressure ventilation is provided, except for sleep apnea. Management is highly structure and protocol driven, with very frequent scheduled medical rounds and admissions, discharges, and rounds guided by written checklists. For UC, there is a nursing ratio of approximately 1 nurse to 10 patients, general physicians covering multiple wards, intermittent observations, scheduled patient rounds by the surgical team, and attendance by physicians as needed.

The anesthesiologist and surgeon were aware of the treatment allocation, with clinical care at their discretion. An ARRC physicians received a handover from the anesthesiologist on the patient’s arrival in PACU and commenced management from then, with input from surgery or other specialties as indicated. Patients were reviewed by the ARRC clinician and surgical team the morning after surgery and transferred to the ward for ongoing care unless ICU transfer was deemed necessary. A specialist anesthesiologist provided supervision of ARRC. ARRC was terminated the morning after surgery, with care after that the same between groups.

### Data Collection and Outcomes

Inpatient data were collected from the statewide electronic medical record (EMR) and electronic anesthetic records by ARRC resident clinicians and a dedicated research nurse, all with specific training in NSQIP scoring and research Good Clinical Practice guidelines. Data after hospital discharge were collected by Good Clinical Practice–trained research staff via the EMR or a phone call with patients or their carers 30 and 90 days after surgery. Data were captured on clinical research forms and subsequently entered into a database.

End points were defined prior to trial commencement. The primary end point was days at home (DAH) at 30 days, the most common period used to examine postoperative outcomes. DAH at 90 days was included to explore a more persistent effect. DAH at 30 days is a relevant end point to consumers and hospitals and known to correlate closely with quality of inpatient care.^18^ Home was defined as the usual place of residence before surgery. Cost-effectiveness analysis, a secondary end point, will be reported separately but uses prespecified outcomes, including time in hospital locations (ARRC, general ward, or ICU) and time spent in posthospital care (residential care, medical practitioner visits, or emergency department visits), as well as mortality. Data were collected on postoperative in-hospital emergencies. The EMR was screened by ARRC physicians out to postoperative day 9 for entries meeting the criteria for a medical emergency response (MER) or rapid response team.^19^ These criteria are widely used, relatively standardized across hospitals, and electronically flagged by the EMR, assisting identification. The criteria used at the Royal Adelaide Hospital are detailed in eAppendix 2 in Supplement 1.

### Sample Size

Sample size estimation for the primary end point of DAH at 30 days was based on data from the feasibility trial, recognizing uncertainty because of low trial numbers and subsequent enhancements to the ARRC model.^8^ Using 2-sample Satterthwaite *t* tests assuming unequal variances, a group allocation ratio of 1:1, mean DAH at 30 days differences of 1.5 days (considered clinically significant to the hospital’s capacity and cost), standard deviations for ARRC/UC of 8.56/9.4, power of 80%, α of .05, and a 2-sided test. The sample size required was 1130 patients.

### Statistical Analysis

As treatment group allocation was not randomized, propensity score (PS) matching was undertaken to mitigate group differences. Multiple variables of relevance to group allocation and outcomes were included in this analysis and are detailed in eAppendix 3 in Supplement 1. The approach used was the nearest neighbor matching with a caliper of 0.2, allowing comparison of each patient’s outcome with that of a patient with similar characteristics.

For the primary end point, DAH at 30 days, a paired *t* test was used to compare the PS-matched groups. For continuous demographic characteristics and secondary end points, the same approach was used for comparing matched data, with McNemar test used for the paired comparisons of binary outcomes, including the incidence of MER-level events.

For reference, the same comparisons were performed on the raw data before matching, but due to skewness of the continuous outcomes, Wilcoxon rank sum tests were used. Fisher exact test was used to compare the data before matching for binary outcomes. Recruitment was paused and a preplanned interim analysis conducted at the 75% recruitment mark, because of the previously mentioned power analysis uncertainty.

A 2-sided *P* value less than .05 was considered significant. Data were captured on clinical research forms and subsequently entered into a database (REDcap version 12.5.16), with all statistical analysis performed using R version 4.2.1 (The R Foundation).

## Results

**Figure 1.**
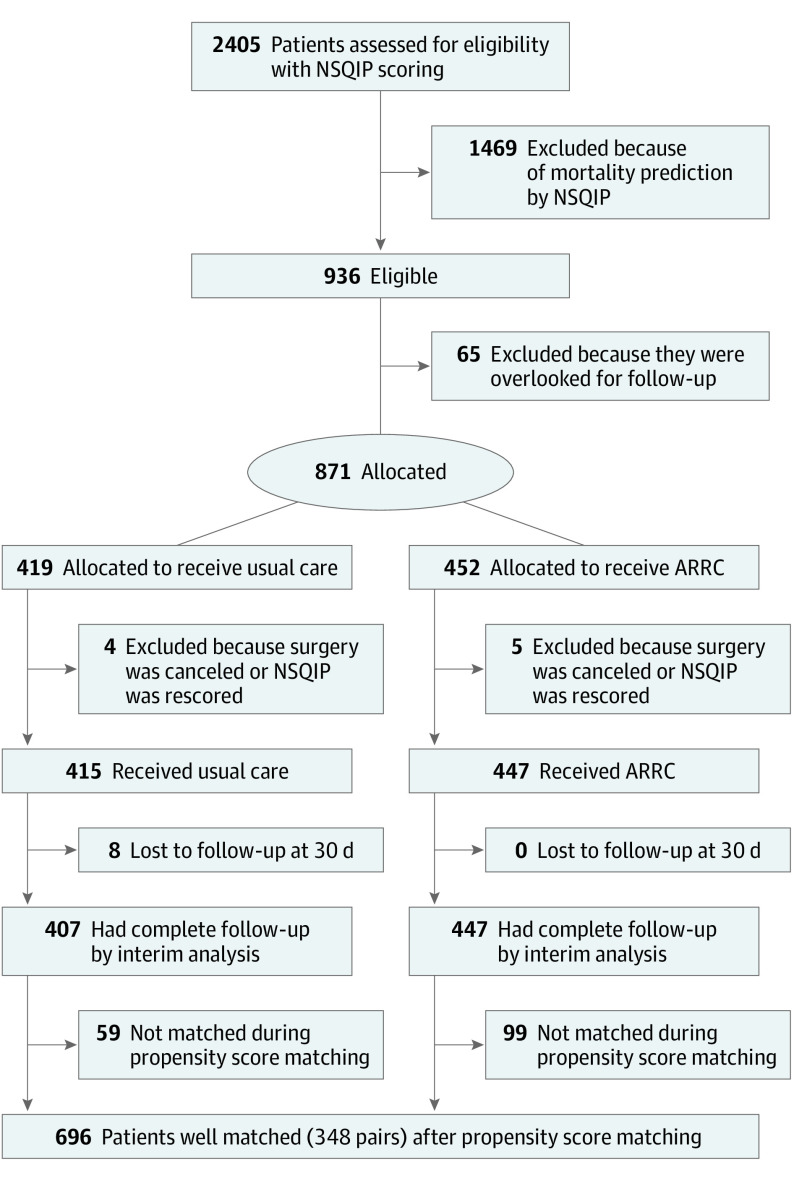
Trial Profile ARRC indicates advanced recovery room care; NSQIP, National Safety Quality Improvement Program.

The trial commenced March 1, 2021, with last patient data collected March 23, 2022. Recruitment was paused November 23, 2021, with an interim analysis conducted once the last patient received their 30-day follow-up. In total, 2405 patients were screened for eligibility with NSQIP, with 1469 not meeting eligibility criteria. Of the 452 allocated to ARRC and 419 to UC, 8 were lost to follow-up at 30 days. The trial profile is presented in Figure 1. At the interim analysis, 407 and 447 patients in the UC and ARRC groups, respectively, had been successfully observed for 30 days. The data safety and monitoring committee recommended the trial be stopped, as the primary end point had reached significance.

The groups’ characteristics are shown in Table 1. The data for all patients showed the groups were well matched for most characteristics, particularly NSQIP outcome predictions relevant to study end points. However, before PS matching, the groups differed in terms of the proportion of elective surgery (more unplanned surgery in the ARRC group) and surgical specialties (more colorectal and gynecology surgery and less orthopedic surgery in the UC group).

**Table 1.** Patient and Procedure Characteristics From All Patients and Propensity Score (PS)–Matched Patients Observed to at Least 30 Days

Characteristic	No. (%)			
	All patients		PS-matched patients	
	Usual care (n = 407)	ARRC (n = 447)	Usual care (n = 348)	ARRC (n = 348)
Patient characteristics				
Age, median (range), y	73 (19-101)	71 (0-97)	72 (19-97)	72 (0-97)
Sex				
Female	195 (47)	202 (44)	163 (46)	157 (44)
Male	212 (53)	245 (56)	185 (54)	191 (56)
Preoperative comorbidities				
Hypertension	269 (66)	309 (69)	238 (68)	240 (69)
Congestive cardiac failure	36 (8.8)	47 (11)	32 (9.2)	34 (9.8)
Ischemic heart disease	100 (25)	129 (29)	95 (27)	96 (28)
Peripheral vascular disease	79 (19)	86 (19)	69 (20)	66 (19)
Diabetes, oral treatment	79 (19)	81 (18)	63 (18)	65 (19)
Diabetes, insulin treatment	69 (17)	72 (16)	58 (17)	60 (17)
Chronic kidney disease	60 (15)	62 (14)	49 (14)	53 (15)
Chronic respiratory disease	128 (31)	162 (35)	121 (35)	125 (36)
Previous stroke/transient ischemic attack	53 (13)	66 (15)	49 (14)	45 (13)
Dementia	23 (5.7)	10 (2.2)	9 (2.6)	10 (2.9)
Recent smoker, last 12 mo	80 (20)	87 (19)	71 (20)	72 (21)
American Society of Anesthesiologists score				
I	0 (0)	2 (0.4)	0 (0)	2 (0.6)
II	43 (11)	65 (15)	40 (11)	43 (12)
III	330 (81)	355 (79)	284 (82)	279 (80)
IV	34 (8.4)	25 (5.6)	24 (6.9)	24 (6.9)
NSQIP risk score predictions				
30-d Mortality, median (range), %	1.7 (0.7-5.4)	1.7 (0.2-5.9)	1.7 (0.7-5.0)	1.6 (0.7-5.9)
Length of hospital stay, median (range), d	5.0 (0.5-10.5)	4.5 (0.5-16.0)	5.0 (0.5-10.5)	4.5 (0.5-11.5)
30-d Readmission rate, median (range), %	8.7 (2.0-24.6)	9.7 (1.9-28.2)	9.0 (2.0-24.6)	9.2 (1.9-28.2)
30-d Serious complications, median (range), %	11.8 (2.5-32.9)	12.5 (3.2-36.9)	11.9 (2.5-32.9)	12.2 (3.2-36.9)
30-d Any complications, median (range), %	13.0 (3.0-39.0)	14.0 (3.0-42.0)	13.0 (3.0-39.0)	13.0 (3.0-42.0)
Surgery characteristics				
Surgery type				
Colorectal	35 (8.6)	61 (14)	35 (10)	43 (12)
Gynecology-oncology	12 (2.9)	26 (5.8)	12 (3.4)	14 (4.0)
Orthopedic	179 (44)	135 (30)	134 (39)	124 (36)
Vascular	98 (24)	122 (27)	90 (26)	92 (26)
Other	83 (20)	103 (23)	77 (22)	75 (22)
Unplanned surgery	251 (62)	207 (46)	198 (57)	185 (53)
Surgery duration, median (range), min	130 (0-783)	169 (27-585)	141 (0-783)	152 (27-535)
Any intraoperative MER-level event >10 min	55 (14)	59 (13)	44 (13)	48 (14)

Abbreviations: ARRC, advanced recovery room care; MER, medical emergency response; NSQIP, National Safety Quality Improvement Program.

PS matching identified 696 patients with a well-matched pair. This PS subset showed close group matching (Table 1). For the PS subset, DAH at 30 days was significantly greater with ARRC, with a mean difference of 1.74 days (95% CI, 0.11-3.36; *P* = .03) in favor of ARRC (Table 2). DAH at 90 days was not significantly different, with a mean difference of 3.88 days (95% CI, −0.40 to 8.17; *P* = .08) (Table 2).

**Table 2.** Patient Outcomes and Locations of Care in All Patients and Propensity Score (PS)–Matched Patients

Outcome^a^	All patients, mean (SD)		Effect size (95% CI)^b^	*P* value^c^	PS-matched patients, mean (SD)		Effect size (95% CI)^b^	*P* value^d^
	Usual care	ARRC			Usual care	ARRC		
DAH at 30 d	15 (11)	17 (11)	2.9 (1.4 to 4.2)	<.001	15 (11)	17 (11)	1.7 (0.1 to 3.4)	.04
DAH at 90 d	62 (31)	68 (27)	6.1 (2.1 to 10)	.002	63 (30)	67 (27)	3.9 (−0.4 to 8.2)	.08
ICU admission, No. (%)	12 (3.0)	22 (4.9)	1.7 (0.8 to 3.8)	.16	10 (2.9)	15 (4.3)	1.5 (0.7 to 3.4)	.42
Length of hospital stay, d	6.8 (9.1)	6.4 (8.3)	−0.5 (−1.7 to 0.7)	.64	6.7 (8.8)	6.3 (8.1)	−0.2 (−1.5 to 1)	.71
ICU rescue from ward, No. (%)	5 (1.2)	6 (1.3)	1.1 (0.3 to 4.6)	>.99	5 (1.4)	6 (1.7)	1.0 (0.3 to 3.5)	>.99
Readmission at 30 d, No. (%)	46 (12)	44 (10)	0.9 (0.5 to 1.4)	.46	40 (12)	31 (9.0)	0.7 (0.5 to 1.2)	.27
Readmission at 90 d, No. (%)	87 (23)	94 (21)	0.9 (0.7 to 1.3)	.61	72 (22)	72 (21)	1.0 (0.7 to 1.4)	.85
Days in hospital at 30 d	8 (8)	7 (7)	−0.9 (−1.9 to 0.1)	.38	8 (8)	7 (7)	−0.6 (−1.7 to 0.5)	.27
Days in hospital at 90 d	10 (14)	10 (13)	−1.0 (−2.8 to 0.9)	.65	10 (14)	9 (12)	−0.5 (−2.4 to 1.5)	.64
Readmissions at 30 d	0.14 (0.4)	0.12 (0.4)	−0.02 (−0.08 to 0.04)	.50	0.15 (0.5)	0.11 (0.4)	−0.04 (−0.11 to 0.03)	.23
Readmissions at 90 d	0.36 (0.9)	0.29 (0.7)	−0.06 (−0.17 to 0.04)	.67	0.36 (0.9)	0.28 (0.7)	−0.06 (−0.18 to 0.06)	.32
ED visits at 30 d	0.27 (1.0)	0.18 (0.4)	−0.09 (−0.20 to 0.02)	.50	0.29 (1.1)	0.16 (0.4)	−0.13 (−0.26 to 0.00)	.04
ED visits at 90 d	0.51 (1.4)	0.38 (0.8)	−0.13 (−0.29 to 0.02)	.37	0.53 (1.5)	0.35 (0.7)	−0.15 (−0.33 to 0.02)	.09
GP visits at 30 d	0.9 (1.7)	1.0 (1.3)	0.1 (−0.1 to 0.3)	.10	0.9 (1.7)	0.9 (1.4)	0.0 (−0.2 to 0.2)	.89
GP visits at 90 d	2.8 (3.2)	2.8 (2.6)	0.0 (−0.4 to 0.4)	.29	2.7 (3.2)	2.7 (2.5)	0.0 (−0.4 to 0.5)	.97
ED presentation at 30 d, No. (%)	71 (18)	72 (16)	0.9 (0.6 to 1.3)	.58	61 (18)	52 (15)	0.8 (0.5 to 1.2)	.34
ED presentation at 90 d, No. (%)	114 (30)	118 (27)	0.9 (0.6 to 1.2)	.39	94 (28)	87 (26)	0.9 (0.6 to 1.2)	.33
30-d Mortality, No. (%)	10 (2.5)	5 (1.1)	0.4 (0.1 to 1.4)	.19	9 (2.6)	5 (1.4)	0.5 (0.2 to 1.6)	.42
90-d Mortality, No. (%)	19 (4.9)	11 (2.5)	0.5 (0.2 to 1.1)	.09	18 (5.4)	11 (3.2)	0.3 (0.1 to 1.0)	.25

Abbreviations: ARRC, advanced recovery room care; DAH, days at home; ED, emergency department; GP, general practitioner; ICU, intensive care unit.

^a^ At 30 days, there was complete data for DAH and days in hospital, no mortality data for 14 patients, and all other outcomes missing for 8 patients. At 90 days, there were 23 patients missing DAH, days in hospital, and mortality data, with 29 patients missing data for all other outcomes. ICU admission information was missing for 1 patient, and length of hospital stay was missing for 9 patients. These patients were therefore excluded from these calculations.

^b^ Mean differences for numerical outcomes and odds ratios for dichotomous outcomes.

^c^ Test results based on Wilcoxon rank sum tests for numerical outcomes and Fisher exact tests for dichotomous outcomes.

^d^ Test results based on paired *t* tests for numerical outcomes and McNemar test for dichotomous outcomes.

**Figure 2.**
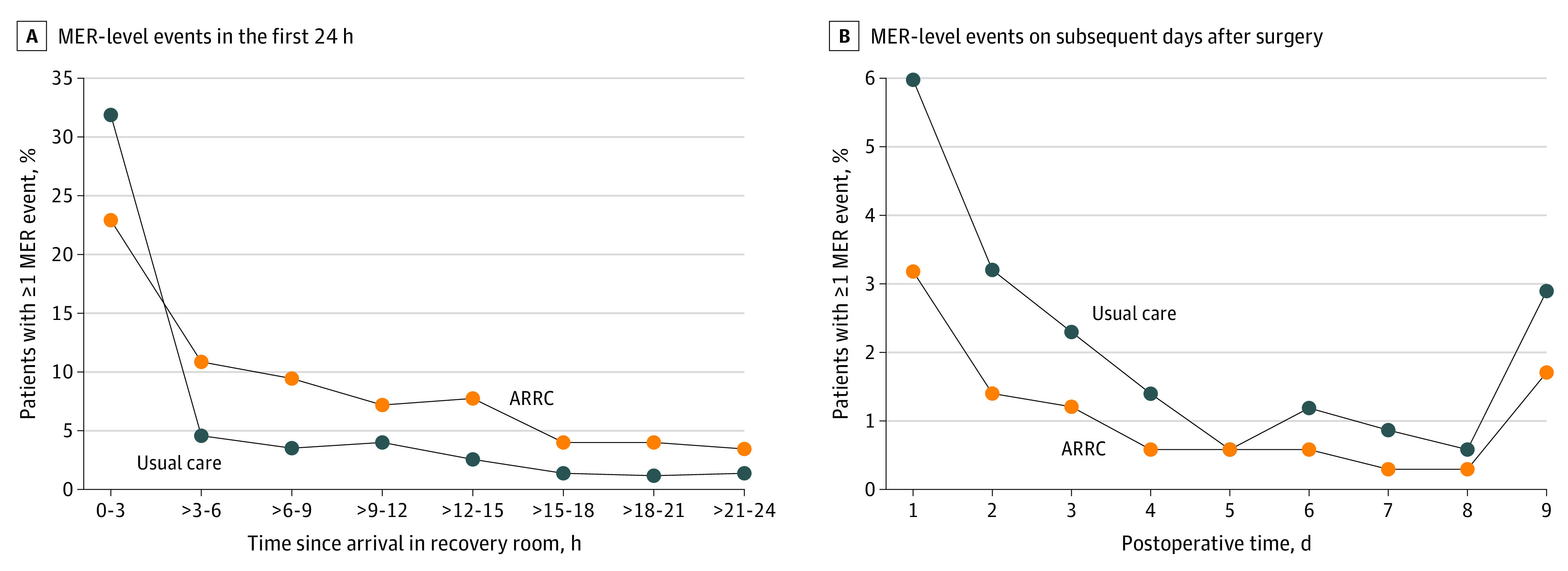
Medical Emergency Response (MER)–Level Events After Surgery Among Patients Receiving Advanced Recovery Room Care (ARRC) or Usual Care A, MER-level events in the first 24 hours after surgery among propensity score–matched patients receiving ARRC or usual care. B, MER-level events up to postoperative day 9 after return to the ward among propensity score–matched patients who received ARRC or usual care in the first 24 hours. Events were more commonly detected in patients who received ARRC in the first 24 hours (*P* < .001) and subsequently less common (*P* = .03).

There were between-group differences in MER-level complications after PS matching. In the first 3 to 24 hours postoperatively, when care was either in the ward or ARRC (for the first 3 hours, both groups were in PACU), MER-level complications were detected approximately twice as frequently in the ARRC group than the UC group, with a mean difference of 8.6% (95% CI, 4.2-13.0; *P* < .001) (Figure 2). In contrast, from day 2 until day 9, when patients from both groups were managed on hospital wards, MER-level complications occurred approximately half as frequently in those who had received ARRC, with a mean difference of −3.7% (95% CI, −7.3 to −0.3) (Figure 2). Hospital MER-level triggers and their group profiles are displayed in Table 3, with a predominance of cardiorespiratory events.

**Table 3.** Frequency of Medical Emergency Response (MER)–Level Triggers in Propensity Score–Matched Patients

MER-level trigger	No. (%)^a^			
	First 24 h		Postoperative days 2-9	
	Usual care	ARRC	Usual care	ARRC
Systolic blood pressure	66 (19.)	96 (27.6)	20 (5.7)	12 (3.4)
Heart rate	10 (2.9)	17 (4.9)	6 (1.7)	3 (0.9)
Saturation	40 (11.5)	36 (10.3)	13 (3.7)	6 (1.7)
Respiratory rate	10 (2.9)	6 (1.7)	4 (1.1)	2 (0.6)
Sedation score	30 (8.6)	18 (5.2)	1 (0.3)	0 (0)
Code blue	1 (0.3)	3 (0.9)	2 (0.6)	0 (0)
Other	7 (2.0)	5 (1.4)	11 (3.2)	3 (0.9)

Abbreviation: ARRC, advanced recovery room care.

^a^ Patients received ARRC or usual care within the first 24 hours and then returned to the ward for postoperative days 2 to 9.

Other postoperative events and predefined end points are displayed in Table 3. There was a statistically significant decrease in emergency department visits but not the other secondary end points of hospital stay, days in hospital, readmissions, and utilization of general practitioners; however, the trial was not powered for these. Mortality rates at 30 days were consistent with NSQIP predictions of approximately 2% in the UC group.

## Discussion

In this study, medical emergencies were detected more often in the ARRC environment during the first day and night after surgery than on the ward where these patients had usually been managed. After discharge from ARRC, when both groups receive the same care, the incidence of these emergencies was lower in the patients who had received ARRC care out to postoperative day 9. This was associated with increased DAH at 30 days, an end point known to be closely correlated with inpatient quality of care.^18^ ARRC now has 10 beds at Royal Adelaide Hospital, based on internal analysis of trial data and the impact on hospital capacity.

Our study specifically targeted medium-risk patients, the population that could directly benefit from an ARRC model yet for whom ward management has been standard of care. A simple example of the future challenge of medium-risk surgical patients is provided by a common operation, a Hartmann procedure, and NSQIP risk calculator predictions.^17^ For someone aged 70 years with hypertension and diabetes, compared with a healthy person aged 60 years, predicted risks of 30-day serious complications, hospital stay, and death increase approximately 3-fold, 2-fold, and 40-fold, respectively,^20^ bringing them into the medium-risk range. By 2030, there will be 750 000 more patients 75 years and older presenting for surgery each year in Britain alone,^21^ with many falling into the medium-risk range. Specifically, low-risk patients may not require enhanced monitoring and specialized medical and nursing staff, and high-risk patients are currently catered for through the ICU-type system. With population aging, there will be proportionally more patients entering the medium-risk category who may require enhanced care during the early phase of recovery but not necessarily ICU management. This has cost implications, as the ARRC model will be more expensive than traditional ward care but less expensive than ICU management. A health economic analysis will be reported separately.

Many clinical trials in recent years have examined the impact of single perioperative interventions on both complications and resource utilization, but benefit is often hard to prove in large multicenter trials.^22^ In contrast, the use of bundles of care may more readily show proof of benefit. This is evident, for example, with programs such as enhanced recovery after surgery, which includes numerous interventions before, during, and after surgery and which may have positive impacts on surgical complications and hospital capacity.^23,24^ However, suboptimal compliance with the protocols significantly affects outcomes for enhanced recovery after surgery and other perioperative quality improvement programs.^25,26^

Delivery of a bundle of high-acuity care postoperatively should be superior to ward care because of enhanced capacity to detect deteriorating patients. Further, the ability to provide vasoactive infusions may prevent problems such as progressive hypotension. It follows that early detection of patient problems leads to more rapid institution of remedial therapy, which in turn may reduce complications. The obstacle to expanding high-acuity units is their high cost and paucity of data on impact. This may be partly because of inconsistent referrals or utilization patterns and partly because of high reliance on observational data. Perhaps, as a result of these costs and doubt about efficacy, their availability varies substantially across jurisdictions.^27^

It was noted in 2017 that decreased availability of overnight high-dependency units for all medium-risk patients receiving low-complexity bowel surgery showed a negative impact on patient outcome and hospital stay.^6^ The ARRC model drew on this observation but expanded the cohort to more procedures to examine ARRC’s applicably more broadly, added a slightly wider mortality risk range, and focused on consistency, precision, and compliance through checklists, protocols, and frequent scheduled patient rounds. The impact of high compliance may underpin the larger-than-expected positive outcomes from this trial compared with those predicted by our earlier feasibility trial.^8^ For example, overnight in-house physicians in the ARRC arm appeared more effective at addressing the high incidence of MER-level complications, which continued overnight (Figure 1). As a result, a much higher proportion of patients were ready for ward transfer by the morning than in the feasibility trial. The need for elective ICU transfer on day 1 decreased to around 1.3% from the feasibility trial figure of around 10%.

Hypotension was common and is well known to be associated with cardiac, kidney, and bowel complications.^9^ Enhanced care to reduce hypotension in ARRC includes the early use of vasopressors to immediately treat hypotension, frequent cardiovascular observations and derived parameters from continuous blood pressure monitors, and transthoracic echocardiography to diagnose the cause of hypotension and guide optimal fluid management. These actions may have been relevant to both the subsequent decrease in ward-based MER-level events and the low ICU referral rate on day 1.

DAH at 30 days is closely related to the quality of inpatient care^18^ and is highly valued by consumers.^28^ It is a composite end point influenced by factors such as length of hospital stay, readmission rates, and duration of stay and supported postdischarge care, such as in rehabilitation centers. Further, its association with inpatient bed days is likely to affect hospitals’ capacity and financial performance and, thus, the sustainability of surgical services.

Surgery-related hospital stay (length of stay or unplanned readmissions) is most commonly measured out to 30 days. In this study, DAH at 90 days was also explored because of some evidence that unplanned, potentially preventable surgical readmissions may persist for months.^29^ There was a signal that DAH at 90 days was numerically further improved, although not statistically significantly in the PS-matched analysis. A range of other predefined end points was also examined, providing signals to suggest ARRC may have other benefits. While not significantly different from UC, this may reflect a type II error, as the trial was powered only for DAH at 30 days, and PS matching usually tends to bias toward a null result. It was notable that the largest apparent relative difference in these end points was mortality. Mortality at 30 days was consistent with NSQIP predictions in the UC group but possibly numerically halved at 30 and 90 days in the ARRC group. These findings suggest that follow-up longer than 30 days after surgery may yield important information on patient outcomes relevant to in-hospital care. A larger study is required to explore differences in the secondary outcomes.

### Strengths and Limitations

Study strengths included the prospective design, the number of patients across a range of specialties, strict inclusion and exclusion criteria, a highly structured postoperative unit to optimize compliance, and a range of postoperative end points out to 90 days.

This study has limitations. Group allocation without randomization introduces the risk of confounding. We mitigated confounding through PS matching and reporting the outcomes on the matched data rather than total data. Further, similar group NSQIP predictions on risks and outcomes relevant to study end points also suggest good group matching. The study was underpowered based on the original estimated between-group difference in the primary end point. This is a single-center trial, which may reduce external validity. However, the patient risk profile is similar to that in the work of Swart et al,^6^ with similar findings of decreased complications and hospital utilization with high-acuity care. We did not measure specific complications and therefore cannot directly apply causation to the identification and treatment of MER-level complications. This would require a large trial for meaningful analysis. Rather, we used a composite surrogate that is associated with complications.

## Conclusions

In this study of medium-risk patients, brief high-acuity care in the early postoperative period allowed enhanced detection and management of early serious MER-level complications, which was associated with a decrease in subsequent MER events after discharge to the ward, with increased time spent at home after surgery. Possible other outcomes on health care resource requirements and mortality warrant further exploration.

## Notes

Supplement 1. **eAppendix 1.** Comparisons of the Models of Care, ARRC and Usual Ward Care (UC) **eAppendix 2.** Escalation Criteria at Royal Adelaide Hospital Used to Precipitate a MER Call **eAppendix 3.** Factors Used for Propensity Score Matching, ARRC vs UC

Supplement 2. Data Sharing Statement
