# Supplementary material for: Outcomes of Postoperative Overnight High-Acuity Care in Medium-Risk Patients Undergoing Elective and Unplanned Noncardiac Surgery
Source: JAMA Surg. 2023 May 3;158(7):701–8. doi: 10.1001/jamasurg.2023.1035 (PMC10157507; doi:10.1001/jamasurg.2023.1035)
Supplement: Supplement 1. — eAppendix 1. Comparisons of the Models of Care, ARRC and Usual Ward Care (UC) eAppendix 2. Escalation Criteria at Royal Adelaide Hospital Used to Precipitate a MER Call eAppendix 3. Factors Used for Propensity Score Matching, ARRC vs UC [file jamasurg-e231035-s001.pdf]

Supplemental Online Content

Ludbrook G, Grocott MPW, Heyman K, et al. Outcomes of postoperative overnight high-acuity care in medium-risk patients undergoing elective and unplanned noncardiac surgery. *JAMA Surg*. Published online May 3, 2023. doi:10.1001/jamasurg.2023.1035

eAppendix 1. Comparisons of the Models of Care, ARRC and Usual Ward Care (UC)
eAppendix 2. Escalation Criteria at Royal Adelaide Hospital Used to Precipitate a MER Call
eAppendix 3. Factors Used for Propensity Score Matching, ARRC vs UC

This supplementary material has been provided by the authors to give readers additional information about their work.

© 2023 Ludbrook G et al. *JAMA Surgery*.

eAppendix 1. Comparisons of the Models of Care, ARRC and Usual Ward Care (UC)

	ARRC	UC
Location	Close to the Recovery Room (PACU) and operating rooms – same floor, within 50 metres<br>Single location<br>2-4 beds per patient bay	Single rooms<br>Wards of approx. 16 beds<br>Home wards and outlying wards
Monitoring	Continuous ECG, heart rate and rhythm, respiratory rate, pulse oximetry<br>Intermittent non-invasive blood pressure (NIBP), temperature<br>Optional continuous NIBP or direct arterial monitoring, and derived parameters<br>Optional trans-thoracic echocardiography – 'fastscan' level	Intermittent non-invasive blood pressure (NIBP), heart rate, pulse oximetry, respiratory rate, sedation scores, temperature<br>Capacity for continuous monitoring, if indicated
Respiratory therapy	Spontaneous ventilation<br>CPAP, continue pre-existing home treatment<br>Nasal and mask, low flow oxygen	Spontaneous ventilation<br>CPAP, continue pre-existing home treatment<br>Nasal and mask, low flow oxygen
Staffing, medical	Continuous presence of resident medical officer with anaesthesia experience, 24/7<br>Consultant anaesthetist exclusively present 0800-1800, and exclusively remotely available after-hours<br>Access to acute pain service and other specialties, as needed, including ICU and MER team<br>Hospital-wide and ARRC-specific guidelines for management of a range of issues, including blood pressure, fluids, arrhythmias	Resident medical officers and consultant surgeons for home surgical team in-house during the day<br>Resident surgical medical officers in-house covering approx. 3-4 wards overnight<br>Access to acute pain service and other specialties, as needed, including ICU and MER team<br>Most services utilise ERAS protocols and guidelines<br>Hospital-wide on-line guidelines for specific care
Staffing, nursing	Nurse: patient – 1:2 on average<br>Continuous attendance<br>Capacity for temporary 1:1 if needed	Usually approx. 1:10 after-hours<br>Attendance 4 hourly, on average, or as needed<br>Capacity for 'specialled' care if needed – 1:1
Laboratory	Routine postoperative blood tests at admission, and postoperative day 1 prior to 0800 ward round<br>Ready access to repeated arterial blood gas analysis	Postoperative blood tests as per home team judgement<br>Arterial blood gas analysis – occasional, as needed
Medical rounds	On admission to Recovery Room<br>Strictly hourly by anaesthesia staff for 3 hours, then at least 3 hourly until the morning of postoperative day 1, and as needed<br>Formal anaesthesia consultant-led round the morning of postoperative day 1, accompanied by surgical round<br>Guided by a 19 point checklist – see Ludbrook, Lloyd et al., reference 8.	As per home team judgement and procedures<br>Formal rounds each morning, and thereafter as needed
Specific additional therapy	Access to low dose vasopressor infusion (metaraminol up to 4mg/hr) to maintain MAP > 60-65mmHg whilst diagnosis and optimal fluid management initiated	

© 2023 Ludbrook G et al. *JAMA Surgery*.

eAppendix 2. Escalation Criteria at Royal Adelaide Hospital Used to Precipitate a MER Call

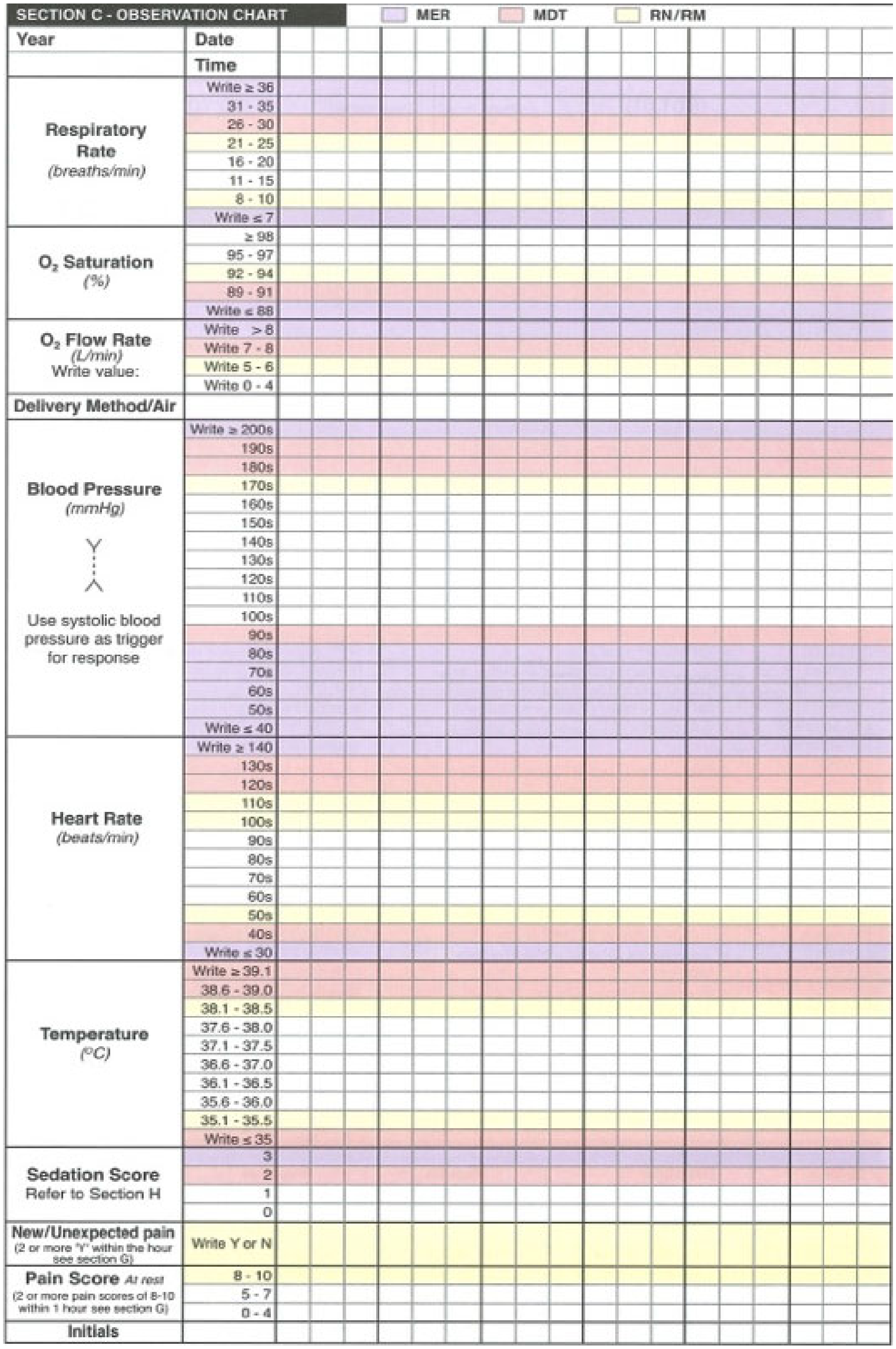

SECTION C - OBSERVATION CHART	MER / MDT / RN/RM
Year	Date
	Time
Respiratory Rate *(breaths/min)*	Write ≥ 36
	31 - 35
	26 - 30
	21 - 25
	16 - 20
	11 - 15
	8 - 10
	Write ≤ 7
O_2 Saturation *(%)*	≥ 98
	95 - 97
	92 - 94
	89 - 91
	Write ≤ 88
O_2 Flow Rate *(L/min)* Write value:	Write > 8
	Write 7 - 8
	Write 5 - 6
	Write 0 - 4
Delivery Method/Air	
Blood Pressure *(mmHg)* Use systolic blood pressure as trigger for response	Write ≥ 200s
	190s
	180s
	170s
	160s
	150s
	140s
	130s
	120s
	110s
	100s
	90s
	80s
	70s
	60s
	50s
	Write ≤ 40
Heart Rate *(beats/min)*	Write ≥ 140
	130s
	120s
	110s
	100s
	90s
	80s
	70s
	60s
	50s
	40s
	Write ≤ 30
Temperature *(°C)*	Write ≥ 39.1
	38.6 - 39.0
	38.1 - 38.5
	37.6 - 38.0
	37.1 - 37.5
	36.6 - 37.0
	36.1 - 36.5
	35.6 - 36.0
	35.1 - 35.5
	Write ≤ 35
Sedation Score Refer to Section H	3
	2
	1
	0
New/Unexpected pain (2 or more "Y" within the hour see section G)	Write Y or N
Pain Score *At rest* (2 or more pain scores of 8-10 within 1 hour see section G)	8 - 10
	5 - 7
	0 - 4
Initials	

© 2023 Ludbrook G et al. *JAMA Surgery*.

eAppendix 3. Factors Used for Propensity Score Matching, ARRC vs UC

The presence or absence of co-morbidities were taken from medical and nursing records for the index surgical admission. Surgical details were taken from the electronic anaesthetic records and EMR

- Current Age (years)
- American Society of Anesthesiologists score (1-4)
- Duration of Surgery (minutes)
- NSQIP-predicted 30-day mortality (%)
- NSQIP-predicted length of stay (days)
- NSQIP-predicted risk of readmission (%)
- NSQIP-predicted risk of serious complications (%)
- NSQIP-predicted risk of any complications
- Unplanned surgery (Yes/No)
- Pre-operative hypertension (Yes/No)
- Pre-operative congestive heart failure (Yes/No)
- Pre-operative ischaemic heart disease (Yes/No)
- Pre-operative peripheral vascular disease (Yes/No)
- Pre-operative diabetes, oral medication only (Yes/No)
- Pre-operative diabetes, including insulin therapy (Yes/No)
- Pre-operative chronic kidney disease (Yes/No)
- Pre-operative chronic respiratory disease (Yes/No)
- Previous transient ischaemic attack or stroke (Yes/No)
- Pre-operative dementia (Yes/No)
- Pre-operative cigarette smoking within last 12 months (Yes/No)
- Colorectal surgery (Yes/No)
- Gynaecology-Oncology surgery (Yes/No)
- Orthopaedic surgery (Yes/No)
- Vascular surgery (Yes/No)

© 2023 Ludbrook G et al. *JAMA Surgery*.
