# Supplementary material for: Outcomes of Postoperative Overnight High-Acuity Care in Medium-Risk Patients Undergoing Elective and Unplanned Noncardiac Surgery
Source: JAMA Surg. 2023 May 3;158(7):701–8. doi: 10.1001/jamasurg.2023.1035 (PMC10157507; doi:10.1001/jamasurg.2023.1035)
Supplement: Supplement 2. — Data Sharing Statement [file jamasurg-e231035-s002.pdf]

Data Sharing Statement

Ludbrook. Outcomes of Postoperative Overnight High-Acuity Care in Medium-Risk Patients Undergoing Elective and Unplanned Noncardiac Surgery. *JAMA Surg*. Published May 03, 2023. doi:10.1001/jamasurg.2023.1035

Data
Data available: No

Additional Information
Explanation for why data not available: Data may be made available on request
